# Autoimmune hemolytic anemia in a patient with chronic lymphocytic leukemia

**DOI:** 10.1002/ccr3.2816

**Published:** 2020-03-27

**Authors:** Samer Al Hadidi, Mark Udden

**Affiliations:** ^1^ Department of Hematology Oncology Baylor College of Medicine Houston TX

**Keywords:** autoimmune hemolytic anemia, chronic lymphocytic leukemia, peripheral blood smear, spherocytes

## Abstract

Peripheral blood smear for patients with CLL and AIHA usually shows lymphoid cells with scant cytoplasm and small round nuclei with condensed chromatin, smudge cells and spherocytes.

## Graphical abstract

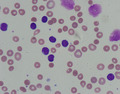

## 1 CLINICAL IMAGE

**Figure 1:**
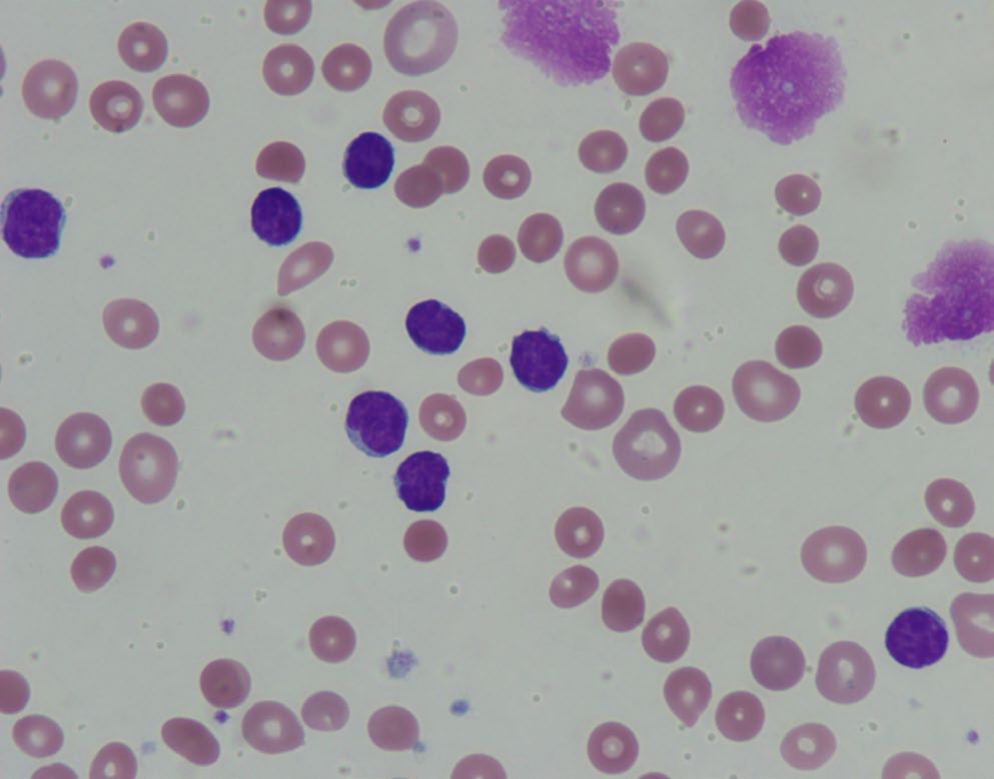
Peripheral smear showing lymphoid cells with scant cytoplasm and small round nuclei with condensed chromatin, smudge cells, and spherocytes

A 60‐year‐old male patient admitted to the hospital with fever, nausea, vomiting, and watery diarrhea of three days. He was diagnosed with chronic lymphocytic leukemia (CLL) 14 years prior to presentation and was treated previously with FCR (fludarabine, cyclophosphamide, and rituximab), and most recently on Ibrutinib. He was also diagnosed with idiopathic thrombocytopenic purpura (ITP) for which he is on eltrombopag. Physical examination showed hepatosplenomegaly. Computed tomography of the abdomen showed enlarging intraabdominal bulky adenopathy. Complete blood count showed leukocytosis (absolute lymphocyte count of 24 K/μL, normal range: 1.32‐3.57 K/μL), macrocytic anemia (hemoglobin of 6.8 g/dL, normal range: 13.7‐17.5 g/dL), and thrombocytopenia (platelets of 32 K/cu mm, normal range: 150‐450 K/cu mm). Laboratory investigations showed hyperbilirubinemia, elevated lactate dehydrogenase, and low haptoglobin. Peripheral smear showed lymphoid cells with scant cytoplasm and small round nuclei with condensed chromatin. Smudge cells were noted with spherocytes (Figure 1). Direct Coombs test (DAT) was negative. Patient was diagnosed with autoimmune hemolytic anemia (AIHA) secondary to CLL and treated with steroids and intravenous immunoglobulin which results in resolution of anemia. Patient was started on Venetoclax for his CLL. Patients with CLL may develop complications related to immune dysfunction resulting in autoimmune disorders and immunodeficiency.^1^ The most common complications are anemia, thrombocytopenia, and infection. Up to one‐third of patients with CLL may develop AIHA over the course of their illness with no relation to treatment modality though a single institution data reported development of AIHA in 4% of patients with CLL.^2^ DAT can be negative in patients with CLL presenting with AIHA.

## CONFLICT OF INTEREST

None declared.

## AUTHOR CONTRIBUTIONS

SA and MU did substantial contributions to conception and design, acquisition, analysis, and interpretation of data, involved in drafting the manuscript and revising it critically for important intellectual content, and gave final approval of the version to be published.
